# Memory Effect in Thermoresponsive Proline‐based Polymers

**DOI:** 10.1002/anie.202209530

**Published:** 2022-10-13

**Authors:** Mostafa Badreldin, Rosanna Le Scouarnec, Sebastien Lecommandoux, Simon Harrisson, Colin Bonduelle

**Affiliations:** ^1^ CNRS Bordeaux INP, LCPO, UMR 5629 University Bordeaux F-33600 Pessac France) E.

**Keywords:** LCST, Polypeptides, Proline, Ring-Opening Polymerization, Temperature Memory

## Abstract

We report that synthetic polymers consisting of *L*‐proline monomer units exhibit temperature‐driven aggregation in water with unprecedented hysteresis. This protein‐like behavior is robust and governed by the chirality of the proline units. It paves the way to new processes, driven by either temperature or ionic strength changes, such as a simple “with memory” thermometer.

## Graphical abstract

Synthetic polymers consisting of *L*‐proline monomer units exhibit temperature driven aggregation in water with unprecedented hysteresis. This protein‐like behavior paves the way to new processes, driven by either temperature or ionic strength changes, such as simple “with memory” thermometer.

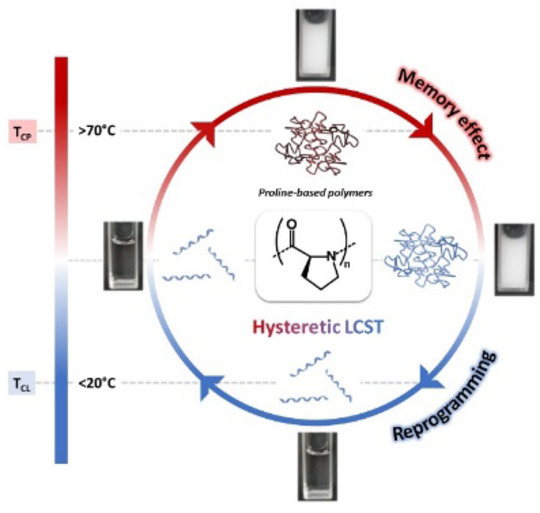

Better understanding the key principles underlying the phase separation of macromolecules is a major challenge. In biology, phase separations of biomacromolecules are at the origin of complex intracellular organization such as the formation of membrane‐less organelles.^[1]^ In medicine, protein phase separations are linked to the formation of pathogenic aggregates associated with neurodegenerative diseases such as Alzheimer's disease.^[2]^ In polymer science, phase separation is an essential property in most formulation processes: for instance, solutions of thermoresponsive polymers undergo phase separation in response to changes in temperature,[^3^, ^4^] an interesting property to develop new solutions in drug delivery, nanotechnology, tissue engineering and biotechnology.^[5]^

Polymers that exhibit a lower critical solution temperature (LCST) in water are miscible with water at all concentrations below the LCST, while above the LCST aggregation may occur to create two immiscible phases in thermodynamic equilibrium.^[6]^ For any specific concentration, the point at which phase separation occurs is known as the cloud point temperature (*T*_CP_, where *T*_CP_≥LCST). Known LCST polymers exhibit small thermal hysteresis in their phase separation behavior, even though this could prove to be a key parameter in designing or programming phase separation driven assemblies (coacervation, etc.).[^7^, ^8^, ^9^] In living systems, such behavior has recently been established for intrinsically disordered proteins: specific recombinantly‐produced sequences of elastin spanning a wide range of molar mass can display hysteretic LCST which hold many promises to engineer advanced materials.^[10]^

In this work, we report a hysteretic thermo‐induced phase separation with simple synthetic homopolymers. These polymers are synthetic polypeptides obtained by ring‐opening polymerization (ROP) of *L*‐proline N‐carboxyanhydride (*L*‐Pro‐NCA).[^11^, ^12^] The ROP of Pro‐NCA monomer has long been limited by its steric hindrance during propagation: the propagating species is a secondary amine and the nucleophilic attack is further weakened by the aggregating cis conformation of the polyproline in organic solvents.^[13]^ Polyproline exhibits unique secondary structures and properties in self‐assembly processes,[^14^, ^15^, ^16^] for example forming thermoresponsive gels,^[17]^ or acting as an antifreezing agent for cell culture.^[18]^

In general, NCA polymerization suffers from significant limitations including tedious monomer purification steps, significant sensitivity to moisture, and a need for toxic solvents such as DMF.^[19]^ Progress has however been impressive: efforts have recently focused on promoting the polymerization with more reactive initiators,[^20^, ^21^, ^22^, ^23^, ^24^] and using aqueous processes including polymerization‐induced self‐assembly.^[25]^ In line with these advances, recent studies have revived the NCA polymerization of polyproline: *J. Kramer* et al. have shown that organometallic catalysis can efficiently achieve previously unexplored high molar masses^[26]^ and *H. Lu* et al. showed that the ROP of Pro‐NCA was water‐enhanced in biphasic mixture containing acetonitrile.^[27]^ Following the aqueous ROP methodology developed in this latest study, we first implemented the polymerization of *L*‐Proline NCA at 0.3 M in a mixture containing 60 % acetonitrile and 40 % water from hexylamine at a monomer/initiator ratio of 50. After a few minutes, the reaction was complete and upon purification, the hexyl‐PLP50 **1** was obtained in high yield (90 %) and characterized by ^1^H NMR, MALDI and SEC analysis (fig S1–3). Although often cited in the literature, the aggregation properties of polyproline with respect to temperature are often poorly described and have mostly been studied with low molar mass oligomers and in non‐aqueous systems.[^28^, ^29^, ^30^] To shed light on this phase transition, we dissolved polymer **1** in water at a concentration of 5 mg mL^−1^ and monitored the solution absorbance by UV spectroscopy at a wavelength of 550 nm while increasing the temperature at a rate of 1 °C/min (Figure 1, red curve).

**Figure 1:**
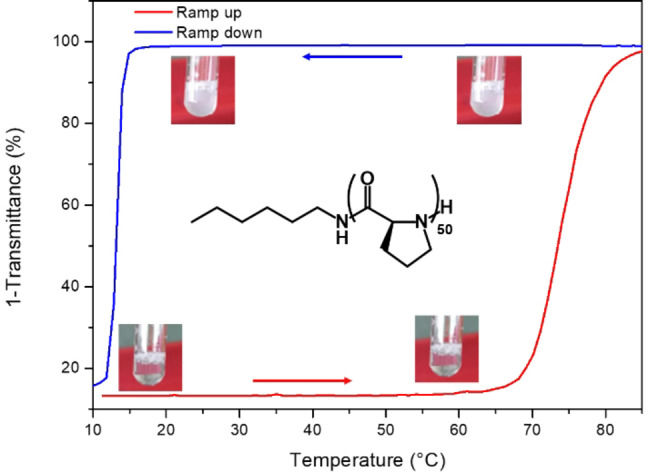
UV/Vis turbidimetry curves for polymer **1** as function of temperature. The sample was heated to 85 °C and aggregated above the *T_CP_* (sharp increase in absorption, red curve). After a 10 min isotherm, the sample was cooled down and the *T_CL_* was only observed at a lower temperature (sharp decrease in absorbance, blue curve) during the cooling process. Absorbance values collected were converted to transmittance for a better visualization of the degree of turbidity.

A cloud point (*T_CP_*) was observed at 67 °C +/−2 °C confirming the LCST aggregation properties of this homopolymer.^[31]^ After a 10‐minute isotherm at 85 °C, the solution was cooled by 1 °C/min to verify the reversibility of the phase transition. Unexpectedly, the clearing point temperature (*T_CL_*) was found at the much lower temperature of 13 °C +/−1 °C, revealing a hysteresis with a breadth of more than 50 °C (Figure 1, blue curve, Figure S4). To evaluate the robustness of this hysteresis phenomenon, we then followed the stability of the aggregation above this clearing point by setting up a new series of analyses with polymer **1** in water at a concentration of 5 mg mL^−1^. For all the solutions, we first monitored the solution absorbance by UV spectroscopy while increasing the temperature at a rate of 1 °C min^−1^ up to a temperature of 85 °C, that was then stabilized for 10 minutes (Figure S5). In a second time, each aqueous solution was cooled down to different temperature values between *T_CP_* and *T_CL_* that was finally stabilized to determine if the aggregates were maintained in time (Figure 2).

**Figure 2:**
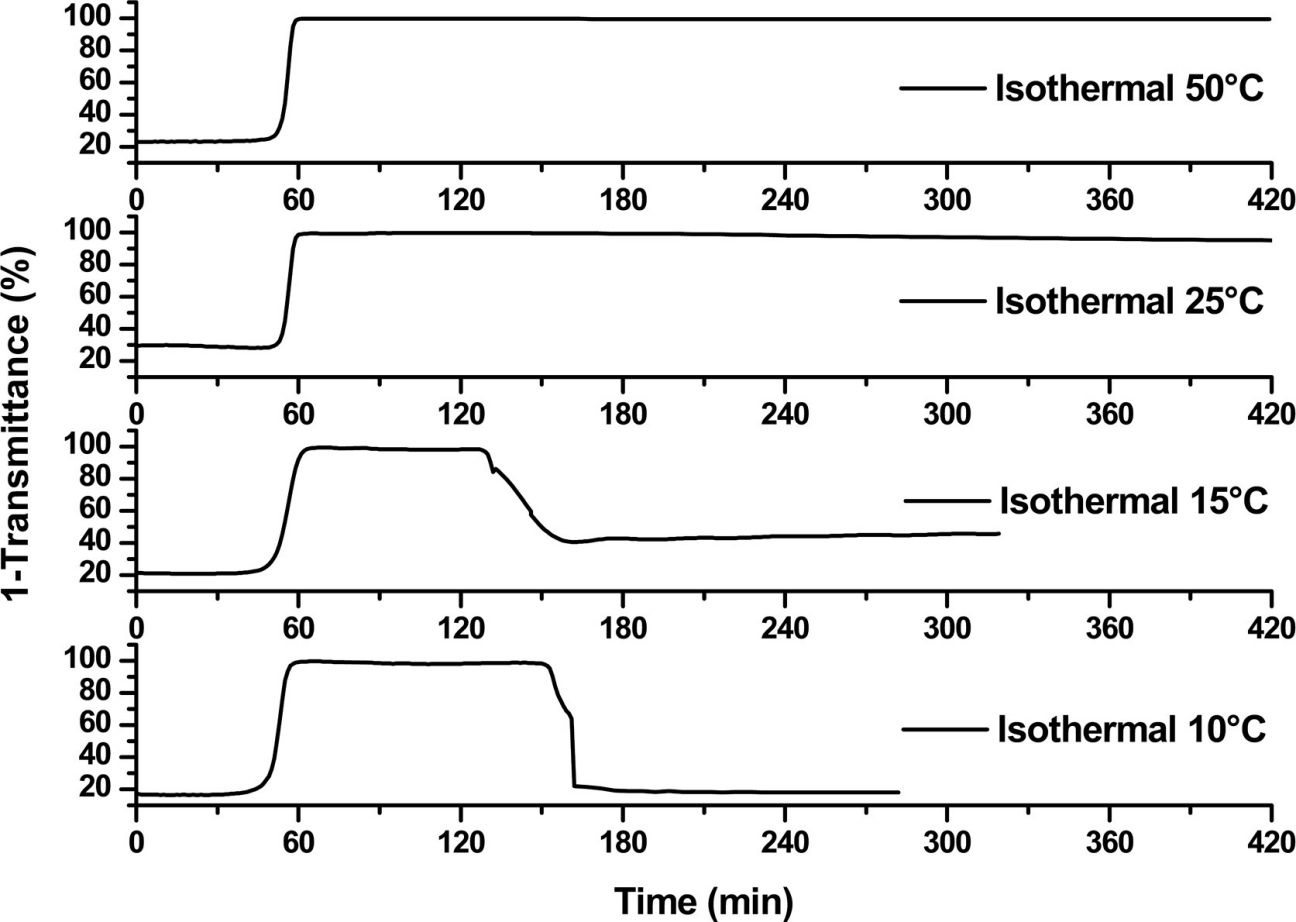
Stability of the aggregation/hysteresis phenomena at different isothermal temperatures. The samples were heated at 1 °C min^−1^ to 85 °C and held for 10 min, during which time aggregation occurred. At t=85 min, the samples were cooled at 1 °C min^−1^ to the indicated temperatures and held at these temperatures with stirring for up to 4 hours. Temperature profiles and isothermal experiments at 37 °C and 60 °C can be consulted in Figure S5.

At isothermal temperatures of 25 °C and above, the turbidity was observed for several hours and even 1 week at 25 °C (Figure 2 and S6). This very encouraging result showed that the “aggregation memory” was sufficiently long and stable to be studied and/or used in a formulation process. On the other hand, cooling to temperatures near the *T_CL_* (15 °C) or lower than the *T_CL_* (10 °C), the resolubilization phenomena was observed.

To further study the hysteresis, several aqueous ROP of Pro‐NCA were carried out in a mixture containing acetonitrile and hexylamine by varying the M/I ratio from 20 to 200 (yield: 61 %–92 %, Table 1). SEC and ^1^H NMR characterizations were performed, and all polymers presented increasing molar masses with controlled dispersities (Table 1 and Figure S7–8). Turbidity measurements by UV spectroscopy were then performed using aqueous solutions of polymers **2**–**7** at a concentration of 5 mg mL^−1^ (Figure 3). These analyses indicated that the hysteresis was increased up to an optimum degree of polymerization (DP) of 200 (hexyl‐PLP20 **7**: hysteresis width of 35 °C, hexyl‐PLP200 **6**: hysteresis width of 60 °C). While *T_CP_* was relatively unaffected by polymer length, the observed differences in hysteresis width were due to the higher clearing temperatures that were observed for shorter polymers (hexyl‐PLP20 **7**: clearing point temperature of 41 °C, Figure 3). *T_CP_* values tended to decrease with increasing polymer concentration in the range 0.5 mg mL^−1^ to 5 mg mL^−1^ (Table S1).

**Table 1:** Polyproline obtained by ROP at different M/I.

Polymer	yield[%]	*Mn* [kg mol^−1^] ^[a]^	*Đ* ^[a]^	*DP* ^[c]^	*T_CP_* [°C] ^[b]^
hexyl‐PLP30 **2**	61	4.0	1.03	30	65+/−3
hexyl‐PLP40 **3**	88	4.1	1.08	41	6+/−2
hexyl‐PLP70 **4**	92	7.8	1.03	70	64+/−4
hexyl‐PLP100 **5**	80	12.4	1.02	120	63+/−1
hexyl‐PLP200 **6**	75	20.1	1.02	207	70+/−3

[a] Absolute number‐average molar mass (*Mn*) and dispersity (*Đ*) determined by SEC using a multi‐angle static light scattering detection. [b] *T_CP_* ( °C) determined using UV/Vis on 5 mg ml^−1^ polymer samples in Milli‐Q water. [c] Polymerization degree determined by dividing the integrals of peaks from the polymer backbone by peaks from the initiator in ^1^H NMR spectra.

**Figure 3:**
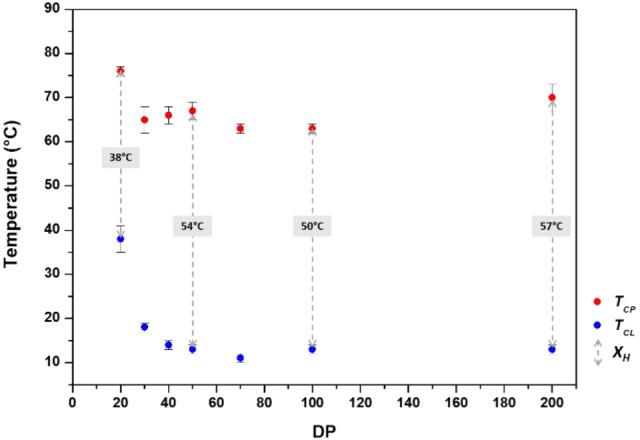
Influence of the polyproline length on the hysteretic LCST behaviour of hexyl‐PLP **1**–**7** at 5 mg ml^−1^. Shorter chains show smaller hysteresis that seems to be mildly affected by chain length at higher DP. *T_CL_* values can be consulted in Table S1.

With poly(*N*‐isopropyl acrylamide), the chemical nature of the end‐groups is an important factor influencing the LCST.^[32]^ The robustness of the hysteresis phenomenon of poly(*L*‐proline) was further studied by using polypeptides prepared by ROP from other initiators. This first included the synthesis of several polymers **7**–**12** at M/I=20 or 70 using small amines with differing hydrophobicity/hydrophilicity such as benzylamine, allylamine, hexylamine and 2‐(2‐aminoethoxy) ethanol. SEC, ^1^H NMR and MALDI‐MS characterizations were performed, and all polymers presented M_n_ values in agreement with the theoretical M/I ratio (Figures S8–10, Table S2). Turbidimetry assays of 5 mg mL^−1^ aqueous solutions of **7**–**12** showed that the polymer extremity had little or no influence on the cloud point, or on the width of hysteresis (Table S2) even at a DP as low as 20 (polymers **7**–**9**). Even PLP‐polyethylene glycol block copolymers, prepared from a polyethylene glycol macroinitiator at M/I=50 with PEG‐NH_2_ 5 kDa (PEG5 K‐PLP50 **13**) and M/I=100 with PEG‐NH_2_ 10 kDa (PEG10k‐PLP100 **14**) showed comparable hysteresis (Table S2, Figure S16–17). SEC, ^1^H NMR and MALDI‐MS characterizations for these polymers can be found in Table S2 and Figures S11–13 of the Supporting Information.

Since polyprolines are known to adopt either of two conformations: an extended, water‐soluble helix designated PLPII or a more compact, hydrophobic helix form known as PLPI, we hypothesized that the aggregation phenomena could be tied to a change in the secondary structure driven by the aqueous solvent.^[26]^ Disrupting the secondary structure by copolymerizing *L*‐ and *D*‐proline at different stoichiometries (poly(prolines) **15**–**20**, Table S3, Figures S17–21) resulted in polymers without thermoresponsive properties, a fact that confirmed the significance of the conformation in the process (Figure S22–S23). A new poly(proline) **21** was then obtained by ROP in pure acetonitrile and under dry conditions (see esi), conditions which lead the polyproline to adopt the PLPI conformation.^[17]^ Figure 4 depicts the CD analysis of **21** in aqueous solution at 25 °C compared to hexyl‐PLP50 **1** before heating (in PLPII conformation) and after heating to 85 °C (Figure 4, red curve).

**Figure 4:**
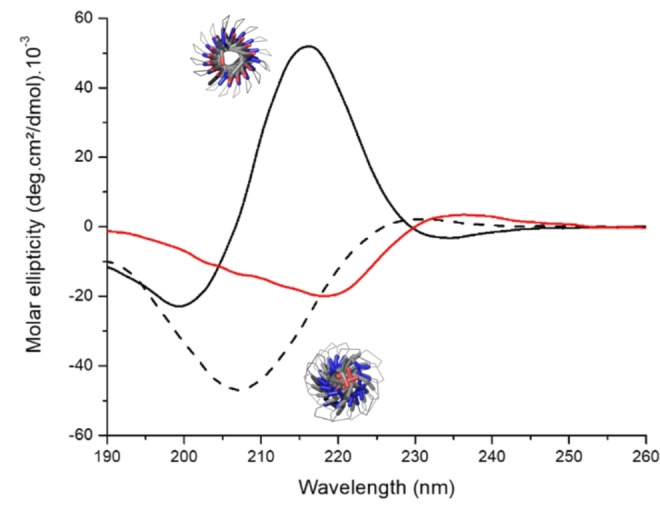
CD spectra of hexyl‐PLP50 **1** before heat treatment (‐ ‐ ‐) in PLPII form (modelized structure, bottom), hexyl‐PLP20 **21**, polymerized in dry ACN (—), right after solubilization in H_2_O in PLPI form (modelized structure, top), and hexyl‐PLP50 **1** after 10 min at 85 °C (—) in an intermediate form between PLPII and PLPI. It should be noted that the concentration is lower here compared to the turbidity tests (cf esi).

The CD trace of the water‐soluble PLPII conformation of hexyl‐PLP **1** (minimum at 208 nm, [θ]=−47(deg cm^2^ dmol^−1^).10^−3^) remained fairly constant from 25 to 75 °C before evolving to a new CD signal at 90 °C that was attributed to a mixture of PLPII and PLPI conformations (Figure S24). LCST hysteresis corroborated a conformational hysteresis: the new CD signal was retained on cooling until about 30 °C before transforming to its original form with further cooling to 15 °C. This change in conformation was also observed by FTIR by monitoring the amide bond stretches of hexyl‐PLP50 **1** in solution before and after thermo‐induced aggregation (Figure S25). Both analyses were in agreement with an incomplete transition to PLPI: indeed, it has been shown that PLPI required several days to be converted to PLPII in water at 4 °C^[26]^ and not instantaneously upon cooling as seen in our results. This corroborates the fact that some trans peptide bonds convert to cis form, an isomerization which can also contribute to the aggregation phenomenon.^[33]^

Hysteresis is a sought‐after memory effect for designing materials but often requires the design of complex systems: this approach includes the use of supramolecular chemistry which has allowed, for example, the fabrication of temperature sensors with memory from specific redox groups.^[34]^ Inspired by these studies, we aimed to develop a simple proof‐of‐concept experiment towards such sensors using our hysteresis phenomenon to memorize the LCST in a temperature range between *T_CP_* and *T_CL_*. With thermoresponsive polymers, the ionic strength of the solution is well‐known to influence the LCST behavior.^[33]^ We therefore studied the influence of different concentrations of NaCl on the thermo‐induced aggregation of hexyl‐PLP70 **4** at a concentration of 5 mg mL^−1^ in water (Figure 5, top). Increasing the ionic strength with NaCl up to 1.5 M significantly reduced the *T_CP_* but had little to no effect on the hysteresis (Figure S26). Beyond 1.5 M, the PLP was insoluble at room temperature due to the salting‐out effect.

**Figure 5:**
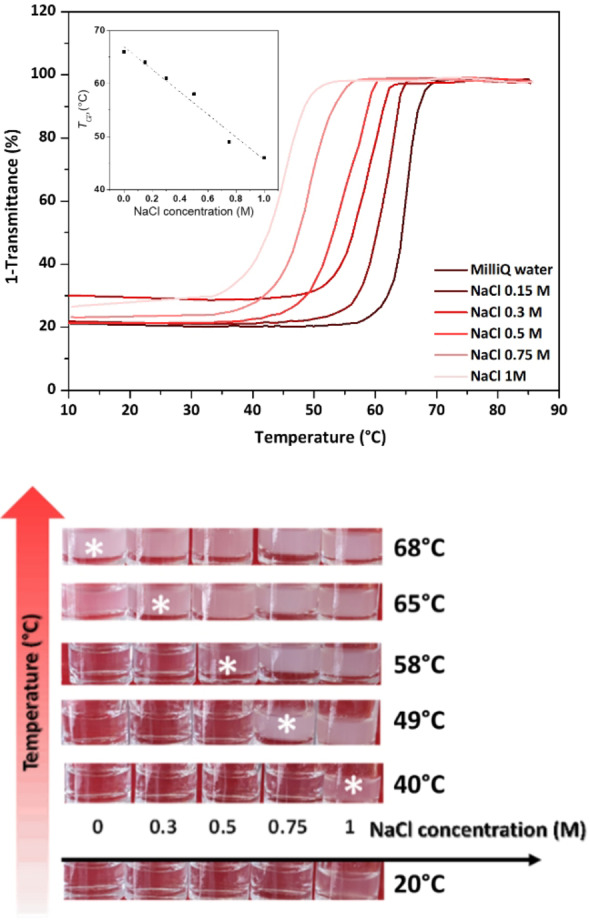
Top: UV/Vis turbidimetry assays showing the effect of salt concentration on the TCP of different solutions of hexyl‐PLP70 4 (5 mg ml^−1^). Plotted in the square is the TCP values depending on the concentration of salt. Bottom: thermometer experience with different solutions of polymer 4 varying only the NaCl concentration. All samples were heated to the temperatures indicated, stabilized for 5 minutes and a picture of the whole series taken at room temperature. The stars highlight the working range of this simple thermometer.

Using 5 solutions containing different salt concentrations and hexyl‐PLP70 **4** at a concentration of 5 mg mL^−1^, we then performed heating cycles at different temperatures. After each heating cycle, the temperature was lowered to room temperature and a picture was taken; as shown in Figure 5 (bottom). These heating cycles revealed the memory kept by the polymer in solution of heating at a temperature above *T_CP_* with an accuracy of few °C. This result is remarkable given the simplicity of the system: it may allow cheap temperature monitoring of many systems or processes in real life applications (pharmaceutical, food processing, etc.) that could be ultimately reprogrammed under *T_CL_*.

In summary, we report that synthetic proline‐based polymers consisting of *L*‐proline monomer units exhibit temperature driven aggregation in water with unprecedented hysteresis. This protein‐like behaviour is robust:^[35]^ it was barely influenced by the concentration and only slightly by the molar mass of the polymers in aqueous solution. However, we found that the thermoresponsiveness and the hysteresis were both governed by the conformation and by the chirality of the proline units. This first‐in‐kind study demonstrates that thermoinduced hysteresis of poly(*L*‐proline) paves the way to new processes, driven by either temperature or ionic strength changes, such as a simple “with memory” thermometer. It establishes the use of synthetic polymers to mimic biologically relevant phase transition highlighting how a rational polymer design influences thermo‐induced aggregation in water and its reversibility.

## Conflict of interest

The authors declare no conflict of interest.

## Supporting information

As a service to our authors and readers, this journal provides supporting information supplied by the authors. Such materials are peer reviewed and may be re‐organized for online delivery, but are not copy‐edited or typeset. Technical support issues arising from supporting information (other than missing files) should be addressed to the authors.

Supporting Information

## Data Availability

The data that support the findings of this study are available from the corresponding author upon reasonable request.
