# Supplementary material for: Memory Effect in Thermoresponsive Proline‐based Polymers
Source: Angew Chem Int Ed Engl. 2022 Oct 13;61(46):e202209530. doi: 10.1002/anie.202209530 (PMC9828171; doi:10.1002/anie.202209530)
Supplement: Supplementary file 1 — Supporting Information [file ANIE-61-0-s001.pdf]

Angewandte Chemie
Eine Zeitschrift der Gesellschaft Deutscher Chemiker

Supporting Information

Memory Effect in Thermoresponsive Proline-based Polymers

M. Badreldin, R. Le Scouarnec, S. Lecommandoux, S. Harrisson, C. Bonduelle**

Table of Contents

Table of Contents 2
Experimental Procedures 2
Part I. Supplementary Experimental Section 2
1. Materials 2
2. Ring opening polymerization (ROP) of N-carboxyanhydrides (NCA)[1][2] 2
Part II. Supplementary Characterization Methods 2
1. Nuclear Magnetic Resonance (NMR) 2
2. Size Exclusion Chromatography (SEC) Analyses 3
3. UV−Vis Spectroscopy 3
4. FTIR spectroscopy 3
5. MALDI-MS measurements 3
6. CD spectroscopy 3
Results and Discussion 4
References 18

Experimental Procedures

Part I. Supplementary Experimental Section

1. Materials

All chemicals and solvents in this work were purchased from Sigma Aldrich, Fluorochem, Acros, TCI, Strem and VWR, and were used without any purification unless otherwise described. Acetonitrile (ACN), was obtained from a solvent system purifier (PureSolv, Innovative Technology), kept under argon atmosphere and freshly used. Milli-Q water was obtained from a Purelab Prima purification system (ELGA) with a resistivity of 18.2 MΩ cm^{-1}. The N-carboxyanhydride monomers were purchased from PMC Isochem, stored at -20 °C under argon atmosphere and weighed in the glove box Jacomex GP13 no. 2675 at the Laboratoire de Chimie des Polymères Organique (LCPO, Bordeaux, France). Dialysis membranes were purchased from SpectrumLabs. Hexylamine, benzylamine, allylamine and 2-(2-amino ethoxy) ethanol were all freeze-thawed and cryo-distilled on the Schlenk line prior to use.

2. Ring opening polymerization (ROP) of N-carboxyanhydrides (NCA)[1][2]

Typical polymerization was adapted from a recent study[1] and goes as follows: *L*-Pro-NCA (100 mg, 71*10-2 mmol, 50 eq) was introduced in a flame-dried Schlenk equipped with a stirrer and suspended in 0.5 ml of dry ACN. Hexylamine (1.43 mg, 1.4*10-2 mmol, 1eq) dissolved in 1.5 ml H_2O: ACN (1:1) at 10°C was added to the first solution to start the ROP under vigorous stirring. The solution became clear quickly and bubbling was observed. FTIR confirmed the reaction completion after 10 minutes by monitoring the disappearance of the bands at 1859 cm^{-1} and 1787 cm^{-1} characteristic of the C=O stretching of NCAs. The reaction mixture was dialyzed against Milli-Q water in a 100-500 Da dialysis membrane to remove all unreacted monomers and solvent before being lyophilized. Yield: 90%, white powder. For polymers prepared in dry acetonitrile, the NCA was dissolved in ACN at 0.1 M and hexylamine was added to start the reaction under inert atmosphere, either under Schlenk line or in glovebox, the solution became milky. Reaction was monitored by FTIR, and at full conversion, acetonitrile was evaporated and polymers were used as such without further purification, Yield: 67%, white powder.

Part II. Supplementary Characterization Methods

1. Nuclear Magnetic Resonance (NMR)

^{1}H NMR 400 MHz spectra were obtained using a Bruker Avance I (Liquid-state 400 MHz NMR spectrometer with 5 mm BBFO probe). Deuterated water (D_2O, Euriso-top, 99.8%) was used as solvent and reference for the lock. The spectra obtained were calibrated using the residual solvent signals (H_2O 4.79 ppm). The signals were categorized as follows: singlet (s), doublet (d), triplet (t), quartet (q), multiplet (m) and broad (br).

2. Size Exclusion Chromatography (SEC) Analyses

Polymer molar masses were determined by Size Exclusion Chromatography (SEC) using an aqueous solvent as the eluent. Measurements were performed on an Ultimate 3000 system from Thermoscientific equipped with diode array detector DAD. The system also includes a multi-angle light scattering detector (MALS) and differential refractive index detector (dRI) from Wyatt technology. Polymers were separated on two TOSOH TSK Gel G4000PWXL and G3000PWXL columns (300 × 7.8 mm) (exclusion limits from 200 Da to 300 000 Da) at a flowrate of 0.6 mL/min. Aqueous solvent composed of acetic acid (AcOH) 0.3 M, ammonium acetate 0.2 M and ACN (H_2O/ACN: 6.5/3.5, v/v) was used as the eluent. Column temperature was held at 25°C. dn/dc measurements were performed on the differential refractive index detector dRI from Wyatt technology by injecting 500 µL of each sample dissolved in the aqueous phosphate buffer at 0.5-5 mg mL^{-1}. The chromatograms were recorded with Chromeleon 7.2 software and Astra 7.1.0 software and analyzed using the latter. dn/dC values calculated were of 0.1616 mg/ml for *L*-Proline homopolymers and copolymers with *D*-Proline and 0.1444 mg/ml for block copolymers with PEG.

3. UV−Vis Spectroscopy

The cloud point temperature (T_{CP}) and the clearing point temperature (T_{CL}) of PLP solutions in pure water were determined by measuring the turbidity at 550 nm between 10°C and 85°C at a 1 °C.min^{-1} scan rate at different concentrations of polymer and salt. Data were collected on a Cary 100 UV−Vis spectrophotometer equipped with a multicell thermoelectric temperature controller from Agilent Technologies. T_{CP} was defined as the temperature at the inflection point of the transmittance-temperature curves during heating ramps and concordantly for T_{CL} during the cooling ramps. A typical experiment followed the following heating program:

1- 10 min isotherm at 10°C
2- 1°C/min ramp to 85°C
3- 10 min isotherm at 85°C
4- 1°C/min ramp to 10°C

Absorbance was plotted in function of temperature, with isotherms' data not collected. Subsequently, absorbance was converted to transmittance for a better visualization of the degree of turbidity and to discriminate between complete and incomplete aggregations. The y-axis in the results is displayed as 1-Transmittance (%) to help the reader relate the soluble and the aggregation form to low and high values respectively.

4. FTIR spectroscopy

The IR spectra were recorded using the FTIR spectrometer (Vertex 70, Bruker) equipped with a temperature control cell, and the samples were measured with the ATR (GladiATR, Pike Technologies) from Fisher technologies performing 64 scans at 2 cm^{-1} in solution at the LCPO (Bordeaux, France). The plate and pressure system of the ATR were equipped with a liquid sample set (Pike Technologies) bought from eurolabo to keep the solvent from evaporating during the heat treatment and thus analyse the sample in the liquid state as other experiments. The raw data were obtained with the Opus7.5 software and processed using the Originlab 2016 software. Samples were dissolved in water and analysed as such on the ATR plate. A background with pure water was run before each sample in the same manner so as not to mask the vibration bands of the C=O bonds between 1500 cm^{-1} and 1700 cm^{-1}.

5. MALDI-MS measurements

MALDI-MS spectra were performed at CESAMO facility (Bordeaux, France) on an Autoflex maX TOF mass spectrometer equipped with a frequency tripled Nd:YAG laser emitting at 355 nm. Spectra were recorded in the positive-ion mode with an accelerating voltage of 19 kV. For MALDI-MS analyses, polymers' deposits were prepared by dissolving 10 mg of polymer in 1 ml of Milli-Q water, 10 mg of NaI in 1 ml methanol and 22 mg of sinapinic acid in methanol. Subsequently 2 µL of polymer solution was mixed with 2 µL of salt solution and added to 20 µL of matrix solution. 1 µL of this mixture was deposited on the MALDI plate and dried.

6. CD spectroscopy

The CD measurements were performed on a JASCO J-815 spectropolarimeter between 190 nm and 260 nm (far-UV). A quartz cell of 1 mm path length (type: 21/10/Q/1) was purchased from Starna Scientific, Ltd. Spectra were recorded at desired temperatures: 20 °C for standard measurements, or a temperature gradient between 10 °C and 90 °C. The measurement parameters were optimized as follows: sensitivity between 5 and 200 mdeg, 0.01 mdeg resolution, 8 seconds response time (Digital Integration Time), 1 nm bandwidth and 10 nm min^{-1} scanning rate. Polymer solutions at a monomer unit concentration of 2.5 µM in Milli-Q water were used for the measurements. For the turbidity tests, the concentration of the samples had to be adapted for this analysis to a concentration of 0.25 mg/mL to avoid flocculation of the polymers. Thus, an optimum concentration was found that neither saturated the signal nor brought the cloud point temperature above reactional conditions.

Results and Discussion

Polymers **1-6** initiated by hexylamine: ^{1}H NMR (400 MHz, D_2O): δ=0.85 (t, 3H; $-CH_3$, hexylamine), 1.26 (6H; $-CH_2-CH_2-CH_2-$hexylamine), 1.45-1.48 (m, 2H; $-C\underline{H_2}-CH_2-NH-$,hexylamine), 2.03 (t, 2H; $-CH_2-C\underline{H_2}-CH_2-$, polymer backbone), 1.80-2.40 (m, 2H; $-C\underline{H_2}-CH_2-CH_2-$, polymer backbone), 3.55-3.90 (m, 2H; $-CH_2-C\underline{H_2}-N-$, polymer backbone), 4.71 ppm (t, 1H; OC-C$\underline{H}$-N, polymer backbone) (Figure S1); MALDI-MS: m/z calcd for $PLP_{60}+Na^+$: 5951.20 $[PLP_{60}+Na]^+$; found 5959.30 (Figure S3).

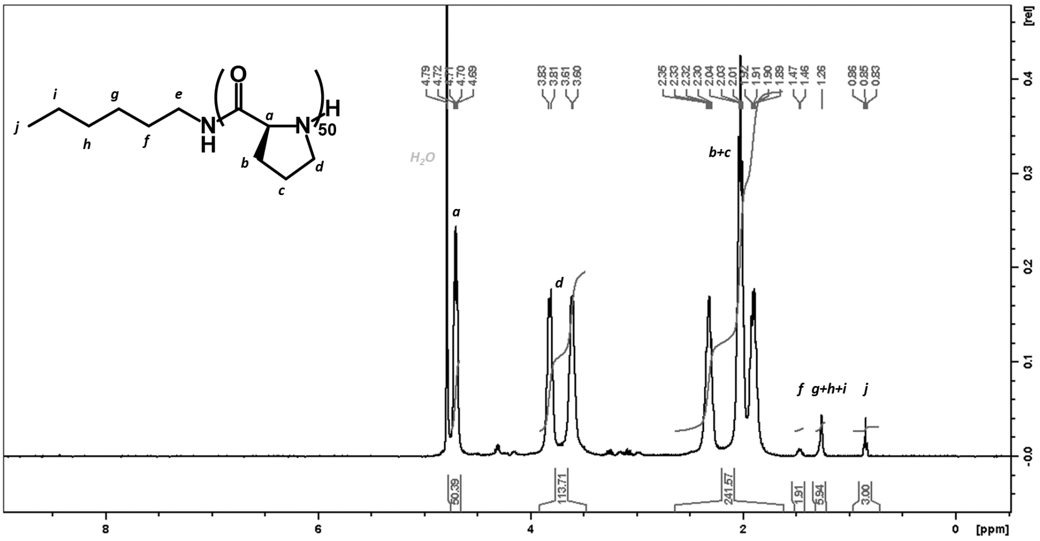

Figure S1. ^{1}H NMR of hexyl-PLP50 **1** in D_2O and polymer scheme. Letters indicate the attribution of peaks to protons.

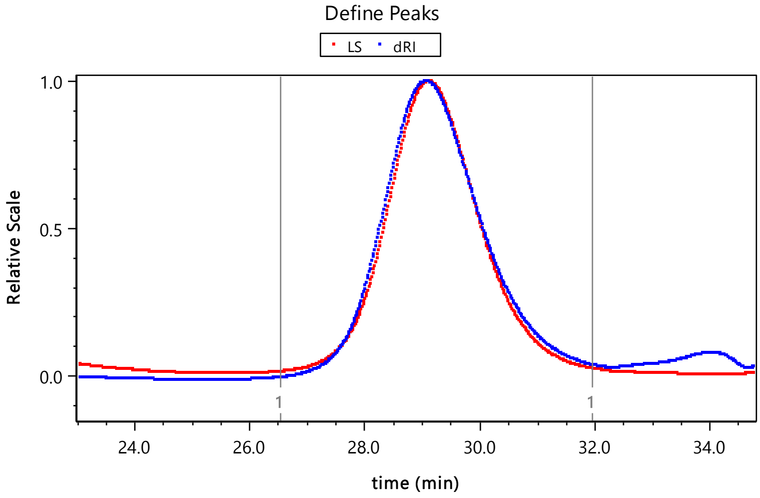

Figure S2. SEC chromatograms of hexyl-PLP50 **1**. In red MALS signal and in blue dRI signal.

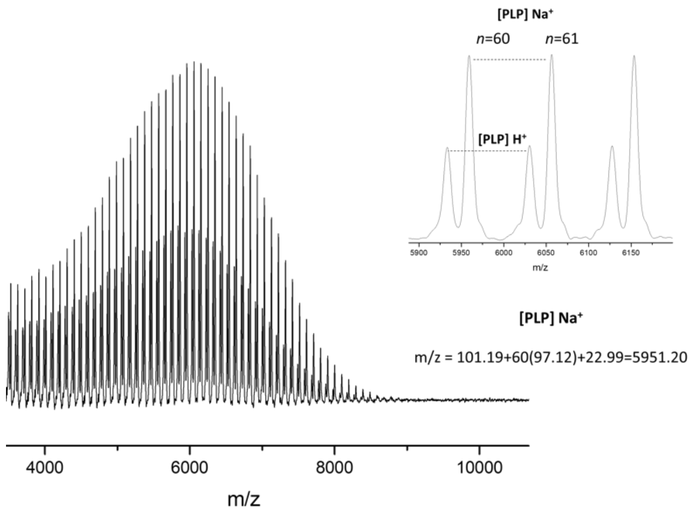

Figure S3. MALDI-MS spectra of hexyl-PLP50 **1**. (On the right) assignment of peaks to proline units along the chain and hexylamine chain end verification.

UV-Vis turbidimetry test, transmission is measured as a function of the temperature in heating and cooling ramps. The derivatives of the curves are used to calculate (T_{CP}), the clearing point temperature (T_{CL}) and the width of hysteresis (X_H).[3]

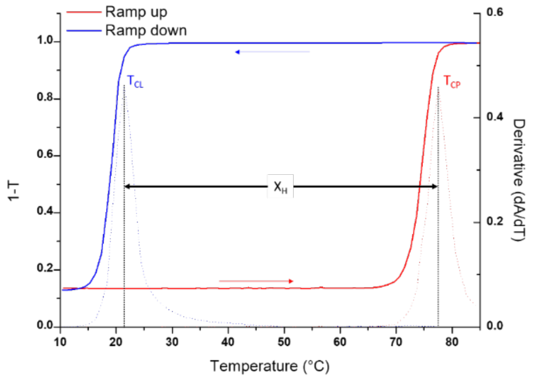

Figure S4. UV-Vis turbidimetry curves for hexyl-PLP50 **1** with characteristic temperatures.

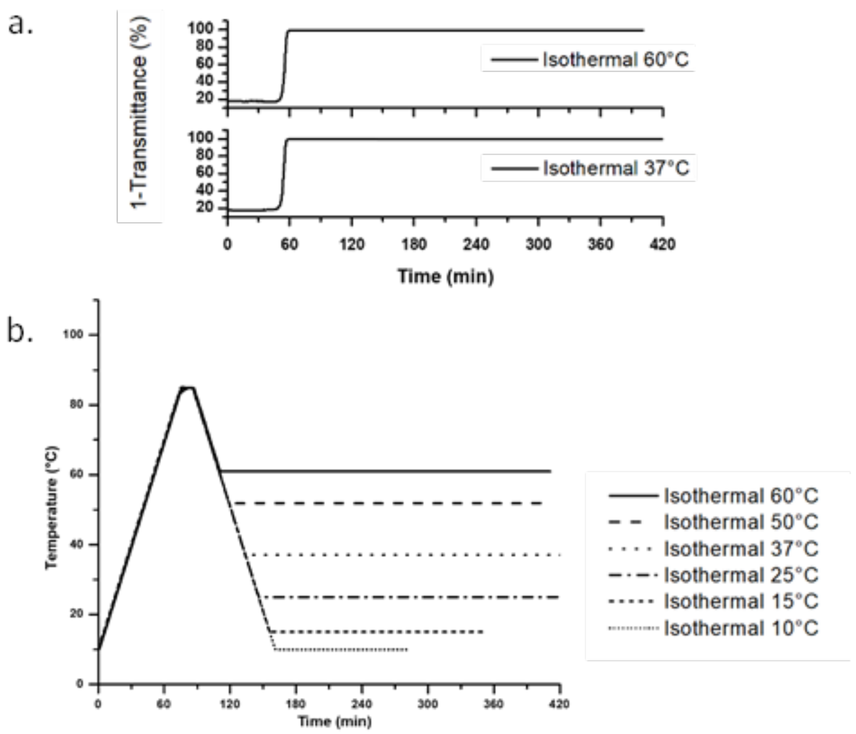

Figure S5. a. Stability of the aggregation/hysteresis phenomena at 60°C and 37°C (isothermal temperatures). **b.** Thermal profiles of all aggregation stability experiments. All samples were heated from 10°C to 85°C at 1°C/min. After aggregation and a 10 min isotherm at 85°C, samples were cooled down by 1°C/min to indicated temperatures (60°C, 50°C, 37°C, 25°C, 15°C and 10°C respectively). Acquisition was continued isothermally for up to 4 hours.

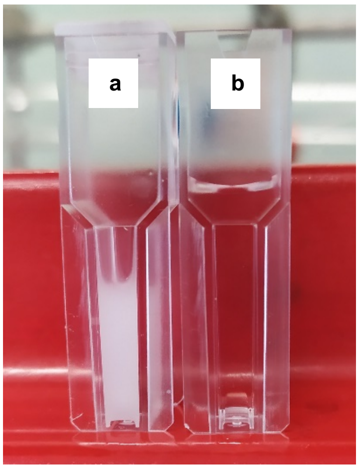

Figure S6. a. The polymer sample after aggregation 1 week after the heat treatment at 25°C. **b.** A sample of the same polymer without any heat treatment solubilized at room temperature.

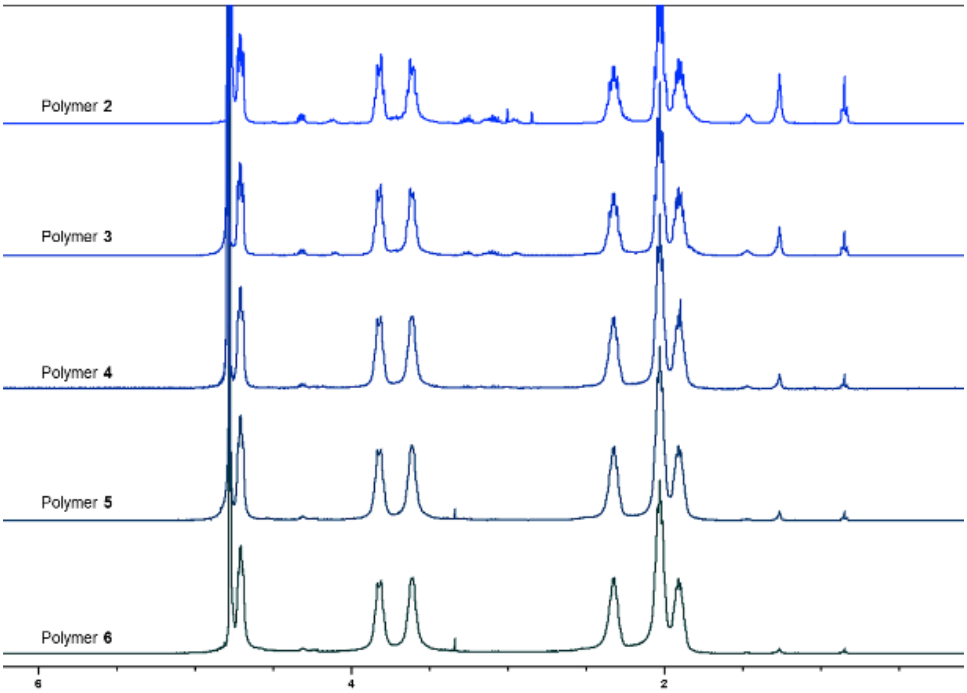

Figure S7. ^{1}H NMR of polymers **2-6** in D_2O.

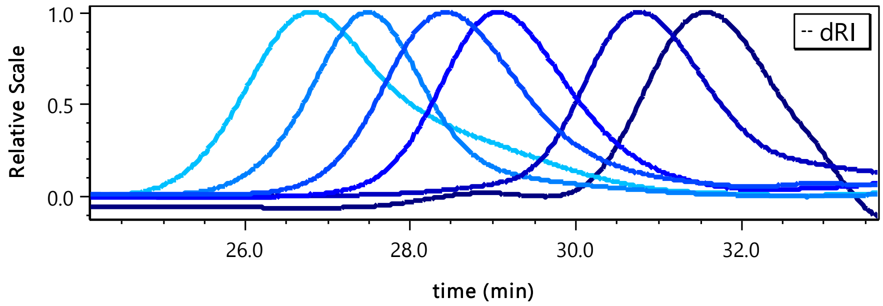

Figure S8. GPC chromatograms of polymers **2-6** in aqueous buffer/ACN.

Table S1. T_{CP} of polymers **1-6** depending on the temperature.

Polymer	T_{CP} (°C) at 0.5 mg/ml [a]	T_{CP} (°C) at 1 mg/ml [a]	T_{CP} (°C) at 2 mg/ml [a]	T_{CL} (°C) at 5 mg/ml [a]
hexyl-PLP50 **1**	>85 [b]	83 +/- 2	77 +/- 1	13 +/- 1
hexyl-PLP30 **2**	>85 [b]	80 +/- 1	73 +/- 3	18 +/- 1
hexyl-PLP40 **3**	83 +/- 2	80 +/- 3	75 +/- 3	14 +/- 1
hexyl-PLP70 **4**	84 +/- 1	77 +/- 2	71 +/- 2	11 +/- 1
hexyl-PLP100 **5**	83 +/- 2	76 +/- 1	71 +/- 3	13 +/- 1
hexyl-PLP200 **6**	>85 [b]	>85 [b]	74 +/- 5	13 +/- 1

[a] Determined using UV-Vis [b] Transitions that were observed to have started before the 10 min stabilization at 85°C, but it was impossible to calculate the derivative of the curve to obtain the temperature value at the inflexion point giving the correct values of T_{CP}.

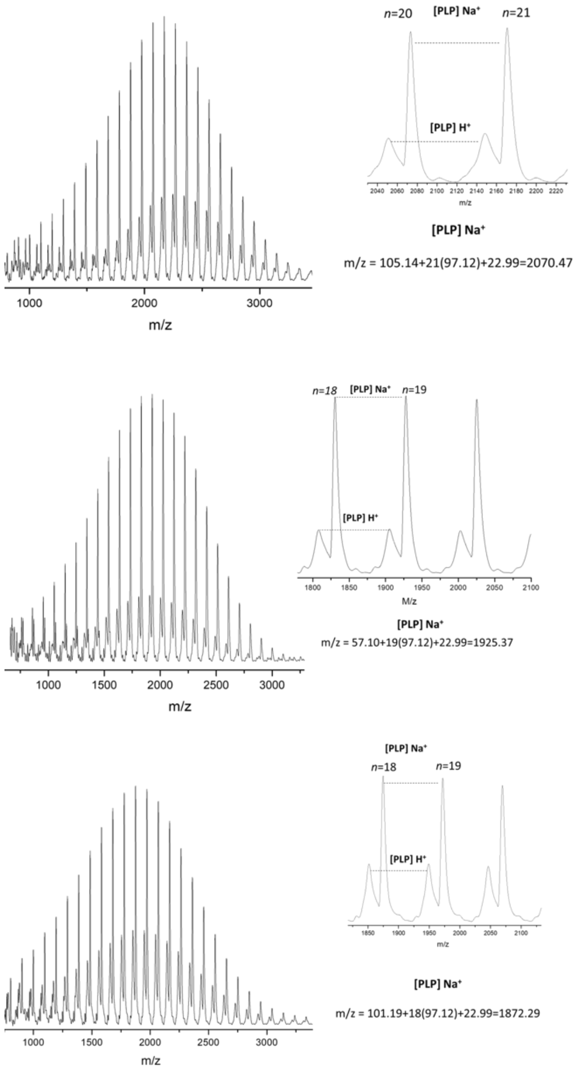

Figure S9. MALDI-MS spectra of polymers **7-9** (from bottom to top)

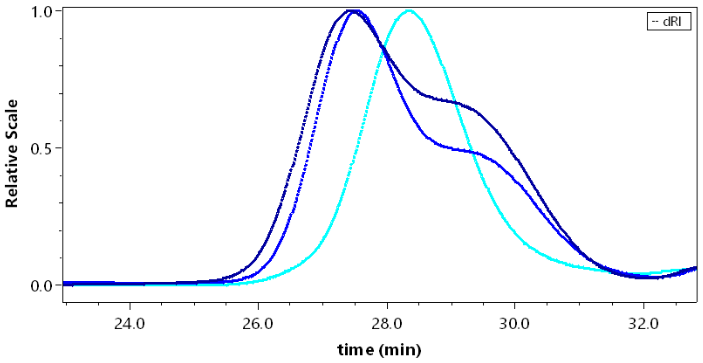

Figure S10. GPC chromatograms of polymers **10**-**11**-**12** in aqueous buffer/ACN.

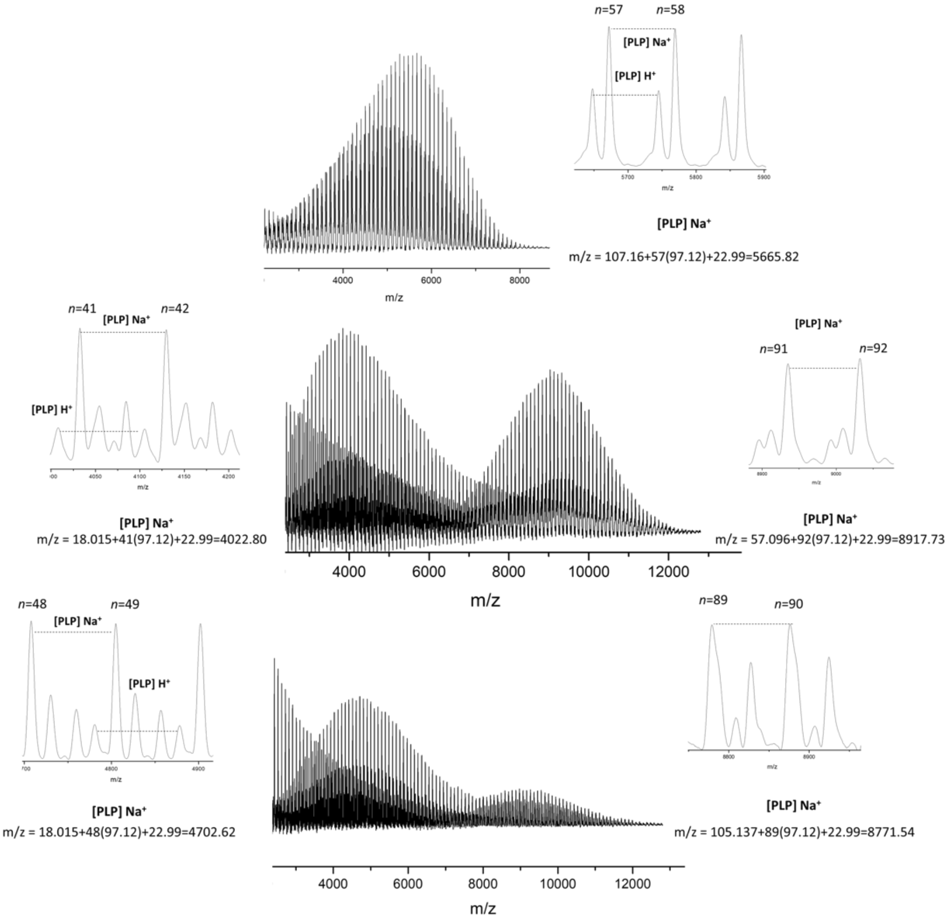

Figure S11. MALDI-MS spectra of polymers **10-12** (from bottom to top)

Polymers **7-9** having small molar masses, SEC analysis was deemed unfit for the calculation of Mn and thus MALDI-MS was used to estimate the Mn and to confirm the initiation by the amine via the end chain(Figure S9). For diglycol-PLP75 **10** and allyl-PLP75 **11**, GPC showed two populations (Figure S10). The second (smallest population) was determined to be polymer chains initiated by water by MALDI-MS via the end-chain group determination (Figure S11). Nonetheless, this did not have any considerable effect on the T_{CP} and the overall thermal behaviour (see Table S2). Only in the case of low DP polymers (**7-9**) we can observe that the hydrophobic hexylamine end chain (hexyl-PLP20 **7**) decreases slightly the T_{CP}. Polymers **10-11** to be compared with hexyl-PLP70 **4** (Table 1 and Table S1).

Table S2. *Tcp* of polymers **7-14** depending on the temperature.

Exp	Initiator	Yield(%)	M_n (kg/mol) [a]	*Đ* [a]	T_{CP} (°C) [c]	T_{CL} (°C) [c]
hexyl-PLP20 **7**	Hexylamine	67	3.9<br>2.0 [b]	1.02	76 +/- 1	38 +/- 3
allyl-PLP20 **8**	Allylamine	55	1.4<br>2.0 [b]	1.04	>85[e]	42 +/- 3
diglycol-PLP20 **9**	2-(2-Amino ethoxy) ethanol	70	2.0<br>2.0 [b]	1.04	>85[e]	35 +/- 3
diglycol-PLP75 **10**	2-(2-Amino ethoxy) ethanol	80	10.3-5.9[d]	1.01-1.03[d]	70 +/- 4	16 +/- 1
allyl-PLP75 **11**	Allylamine	65	10.4-6.7[d]	1.01-1.04[d]	68 +/- 2	14 +/- 1
benzyl-PLP75 **12**	Benzylamine	70	7.5	1.01	65 +/- 2	12 +/- 1
PEG5K-PLP50 **13**	PEG-NH_2 (5kDa)	58	10.9	1.04	84 +/- 1 [d]	25 +/- 3 [d]
PEG10k-PLP100 **14**	PEG-NH_2 (10kDa)	78	19.1	1.04	>85 [d] [e]	22 +/- 2 [d]

[a] Absolute number-average molar mass (M_n) and dispersity (Đ) determined by SEC using a multi-angle static light scattering detection. [b] Average molar mass as determined by MALDI-MS [c] T_{CP} and T_{CL} (°C) determined using UV-Vis on 5 mg/ml polymer samples in MilliQ water. [d] 10 mg/ml polymer samples in Milli-Q water. [e] Transitions that were observed to have started before the 10 min stabilization at 85°C, but it was impossible to calculate the derivative of the curve to obtain the temperature value at the inflexion point giving the T_{CP}. [d] The shouldered peaks shown in Figure S10 were treated by the softwre as two different peaks and thus their M_n and Đ are presented separately here.

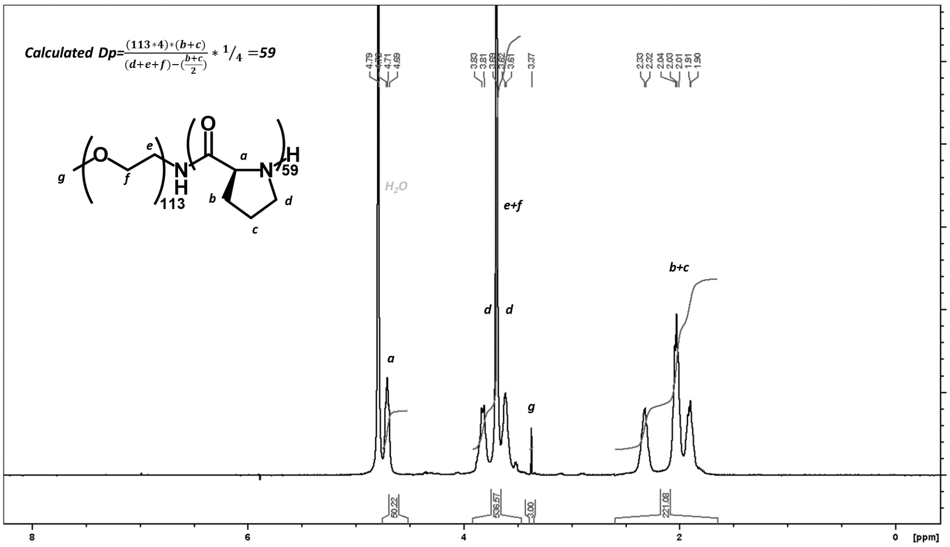

Figure S12. ^{1}H NMR of PEG5K-PLP50 **13** in D_2O. The chemical structure and the DP calculation method are represented on the top left

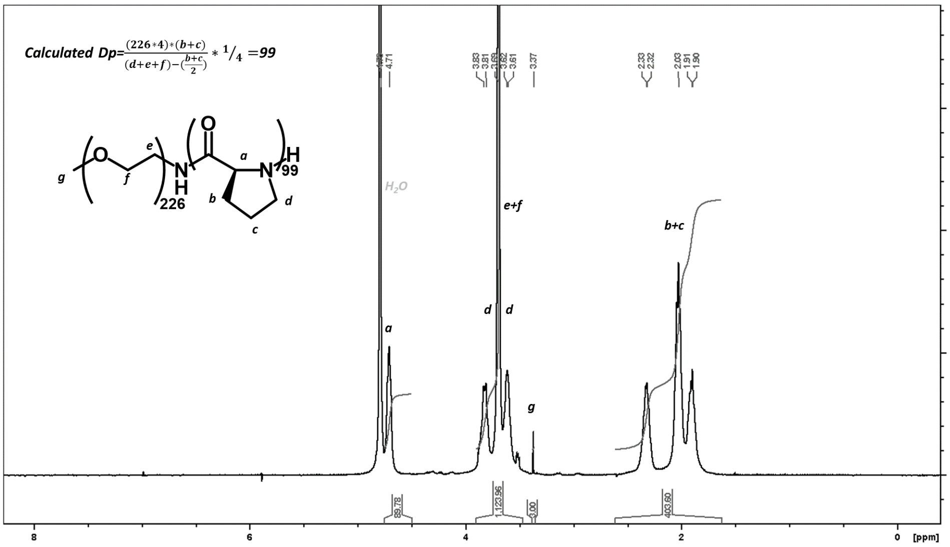

Figure S13. ^1H NMR of PEG10K-PLP100 **14** in D_2O. The chemical structure and the DP calculation method are represented on the top left

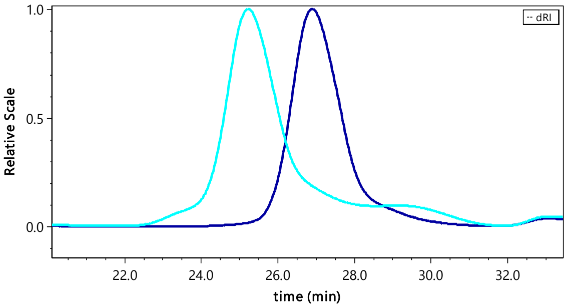

Figure S14. GPC chromatograms of copolymers **13**-**14** in aqueous buffer/ACN.

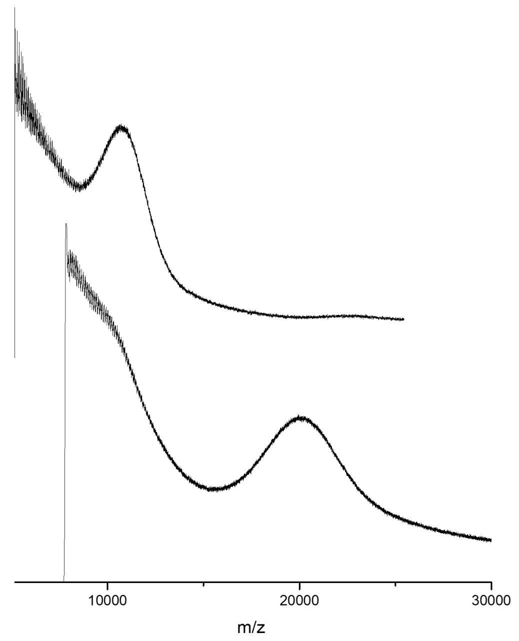

Figure S15. MALDI-MS spectra of polymers **13-14** (from top to bottom)

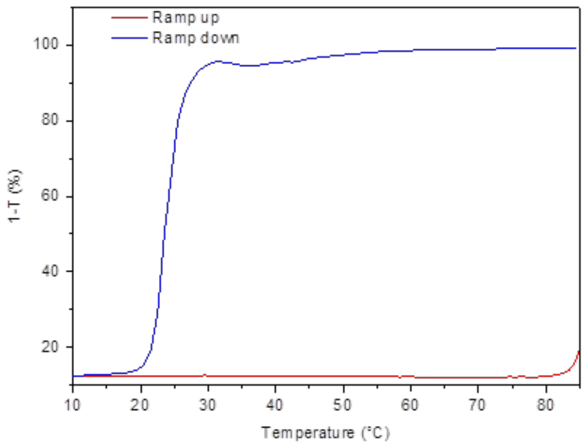

Figure S16. UV-Vis turbidimetry curves for PEG5K-PLP50 **13**.

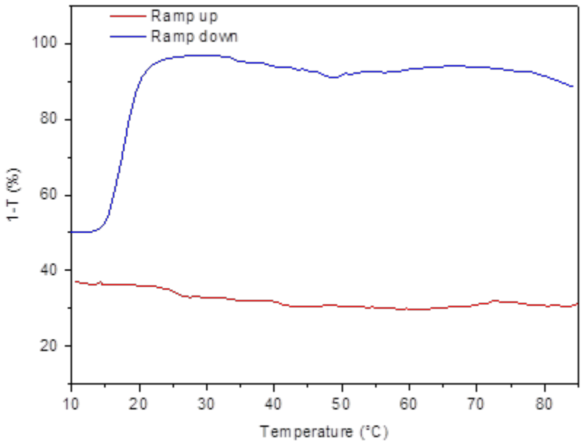

Figure S17. UV-Vis turbidimetry curves for PEG10K-PLP100 **14**.

Table S3. Composition and SEC analysis of copolymers **15-19** and polymer **20.** Hexylamine was used as initiator for all experiments

Polymer	Composition	Yield (%)	M_n (kg/mol) [a]	Đ [a]
hexyl-P(90L/10D)P **15**	90 *L*-Pro/10 *D*-Pro	81	6100	1.18
hexyl-P(80L/20D)P **16**	80 *L*-Pro/20 *D*-Pro	73	6100	1.12
hexyl-P(70L/30D)P **17**	70 *L*-Pro/30 *D*-Pro	85	6000	1.13
hexyl-P(60L/40D)P **18**	60 *L*-Pro/40 *D*-Pro	92	6600	1.17
hexyl-P(50L/50D)P **19**	50 *L*-Pro/50 *D*-Pro	51	6900	1.01
hexyl-PDP100 **20**	100 *D*-Pro	75	11200	1.02

[a] Absolute number average molar mass (M_n) and dispersity (Đ) determined by SEC using a multi-angle static light scattering detection.

Copolymers with a mixture of *L*- and *D*-Proline all showed molar masses lower than theoretical M/I. The *D*-Proline NCA produced hexyl-PDP100 **20** with expected molar mass and controlled dispersity.

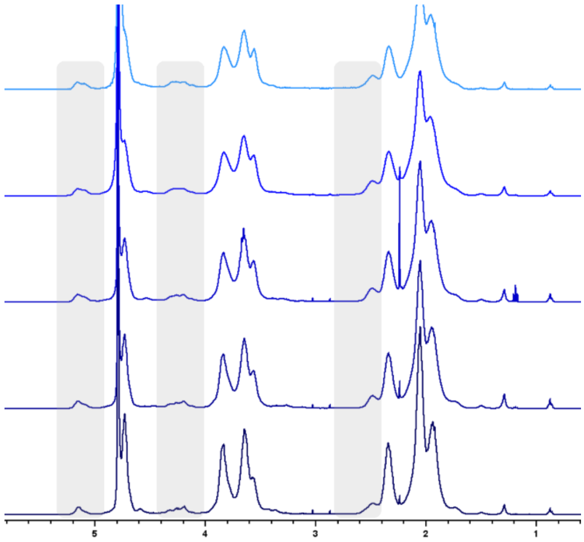

.

Figure S18. ^{1}H NMR of copolymers **15**-**19** (bottom to top) in D_2O. Spectra showed all PLP peaks with some broadening and peaks different from the homopolymer were distinguished (highlighted in grey) and could be attributed to the cis conformation.[4]

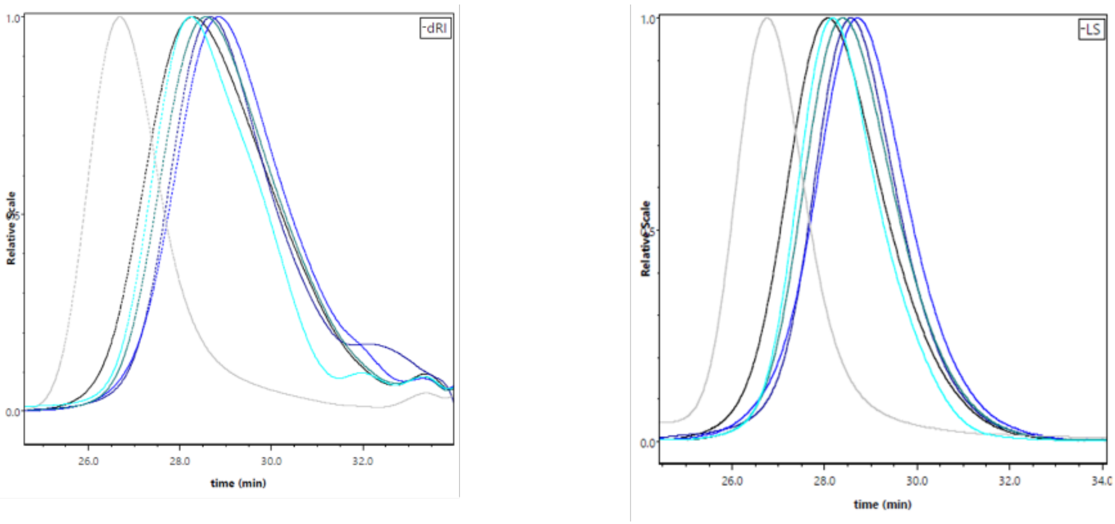

Figure S19. GPC chromatograms of polymers**15**-**19** and hexyl-PDP100 **20** in aqueous buffer/ACN. dRI signal on the left and LS signal on the right

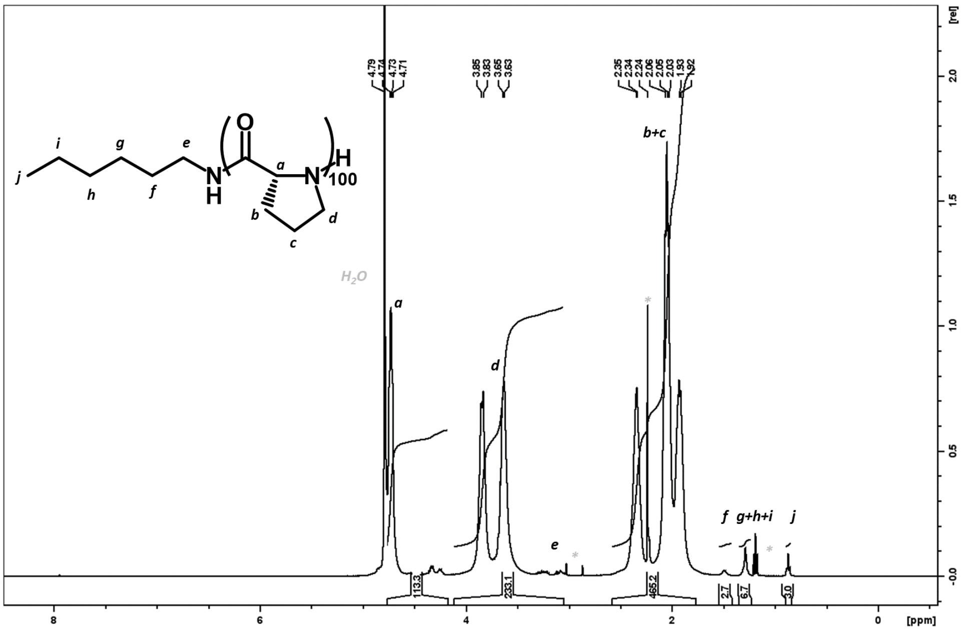

Figure S20. ^{1}H NMR of hexyl-PDP100 **20** in D_2O and polymer scheme. Letters indicate the attribution of peaks to protons. Peaks labelled with * correspond to impurities in the NMR tube.

All polymers containing a mixture of both *D*- and *L*-Proline repeat units showed no thermoresponsive behaviour, confirming our hypothesis of the conformational role on this property. The LCST behaviour of hexyl-PDP100 **20** was comparable to that of its *L* counterpart; polymer **5** (Figure S22).

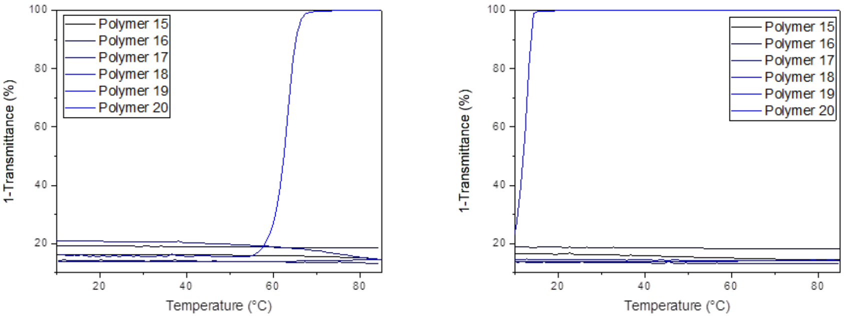

Figure S22. UV-Vis turbidimetry curves for copolymer **15-20**. Ramp up 1°C/min on the left and ramp down 1°C/min on the right. Only hexyl-PDP100 **20** exhibits the hysteretic LCST.

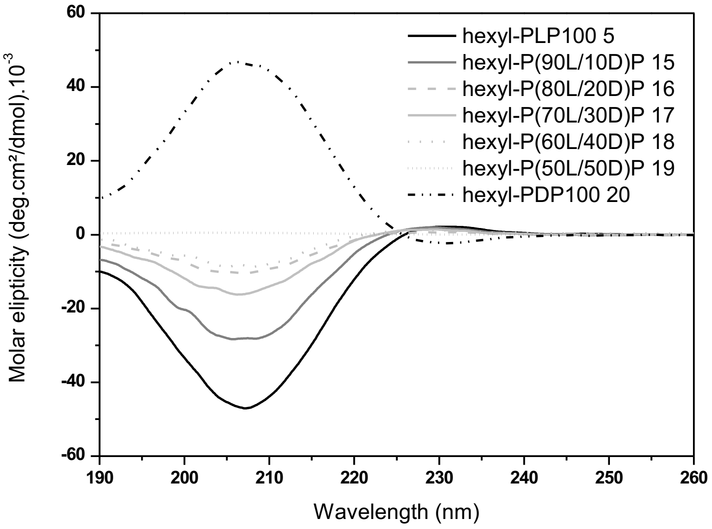

Figure S23. CD spectra of polymers **15-19** and hexyl-PLP **5** at room temperature in milliQ water at a 0.25mg/ml concentration. All polymers show a PLPII conformation except for hexyl-P(50L/50D)P **19** as the signal is canceled out by the presence of an equal quantity of both enantiomers.

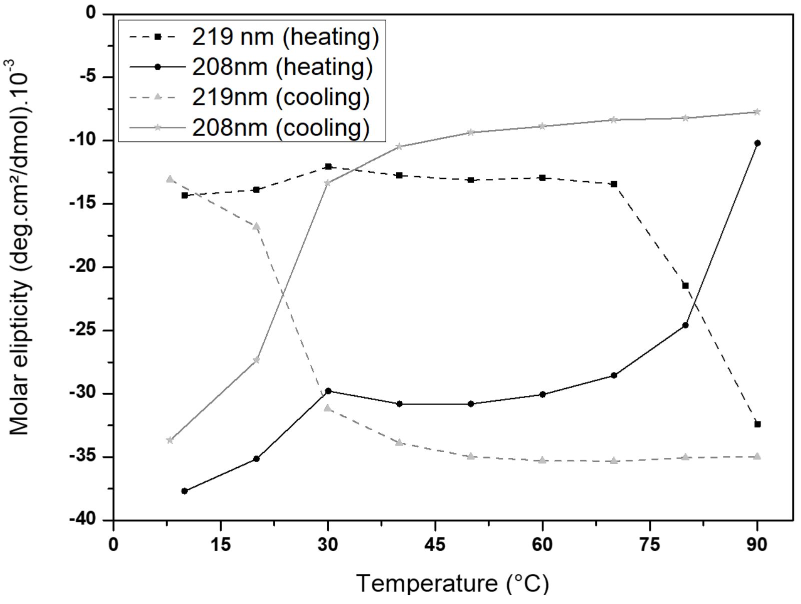

Figure S24. Evolution of CD peaks of hexyl-PLP50 **1** during heating and cooling of a 0.25mg/ml in milliQ water sample. The peak at 208nm (typical of PLPII soluble) sharply decreases in intensity at higher temperatures (80°C) during the heating ramp, while the peak at 208nm (attributed to the aggregated form) sharply increases in its intensity around the same temperature. Both peaks have a constant value in the cooling ramps from 90°C to around 30°C, at which time the tendency inverses showcasing the conformation hysteresis.

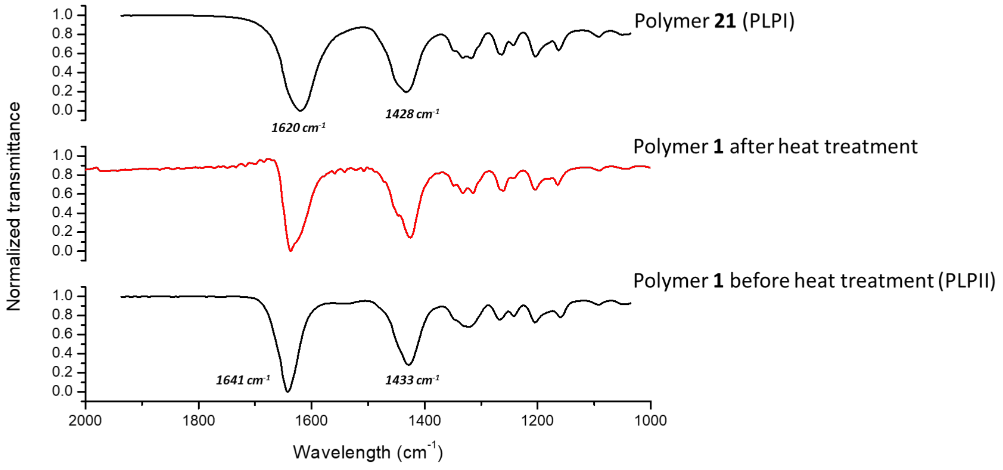

Figure S25. FTIR spectra of polymer solutions in H_2O. hexyl-PLP20 **21** (polymerized in dry ACN) is in PLPI conformation and hexyl-PLP50 **1** in PLPII conformation. hexyl-PLP20 **21** was then precipitated by heating and brought to room temperature (spectra is shown in red). The amide bond stretching wavelength clearly indicate the presence of both conformations.

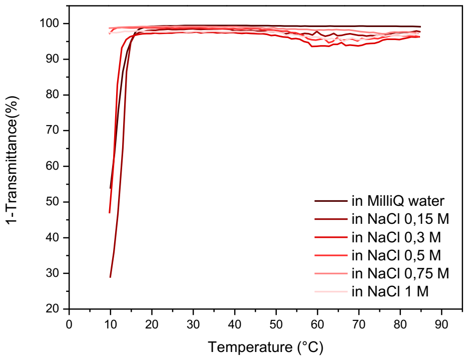

Figure S26. UV-Vis turbidimetry showcasing the effect of salt concentration on the T_{CL} of different solutions of hexyl-PLP70 **4** (5 mg/ml). Completes the temperature cycle of Figure 5. At high concentrations of salt, the T_{CL} was not observed in the time scope of the experiments, but polymers were again soluble after few hours' storage in the fridge at 4°C.

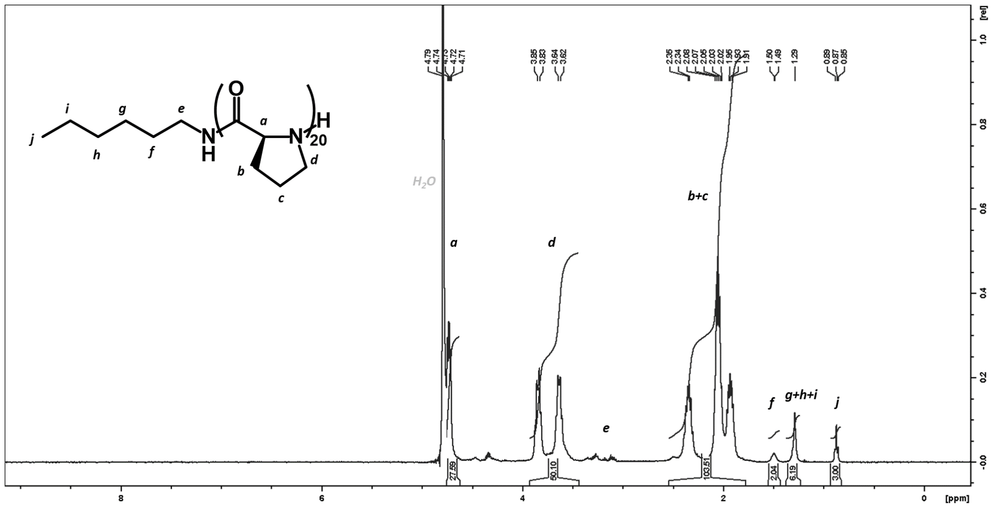

Figure S27. ^{1}H NMR of hexyl-PLP20 **21** in D_2O and polymer scheme. Letters indicate the attribution of peaks to protons.

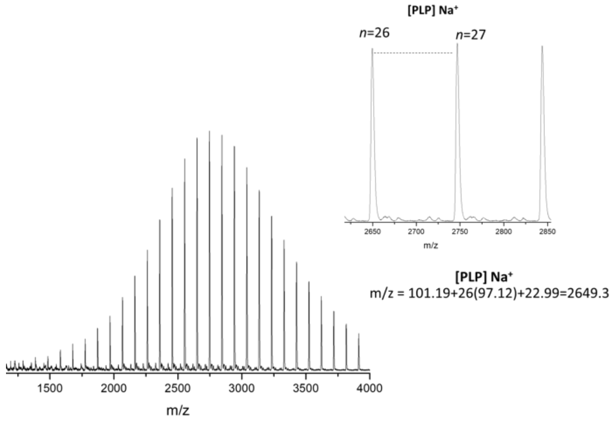

Figure S28. MALDI-MS spectra of hexyl-PLP20 **21**

References

[1] Y. Hu, Z.-Y. Tian, W. Xiong, D. Wang, R. Zhao, Y. Xie, Y.-Q. Song, J. Zhu, H. Lu, *Natl. Sci. Rev.* **2022,** nwac033.
[2] M. Gkikas, R. K. Avery, B. D. Olsen, *Biomacromolecules* **2016**, *17*, 399–406.
[3] Z. Osváth, B. Iván, *Macromol. Chem. Phys.* **2017**, *218*, 1600470.
[4] Y.-Y. H. Chao, R. Bersohn, *Biopolymers* **1978**, *17*, 2761–2767.
